# Tricuspid valve infective endocarditis due to ESBL producing *E.coli* associated with AV block requiring epicardial pacing: A case report

**DOI:** 10.1186/s13019-026-04460-8

**Published:** 2026-06-26

**Authors:** Roben Ohev Shalom, Saro Avedikian, Victoria Watkins, Manraj J. Johal, Varol S. Togay

**Affiliations:** 1 Department of Internal Medicine, Los Robles Health Medical Center, 215 W Janss Road, Thousand Oaks, CA USA; 2 Department of Cardiology, Los Robles Health Medical Center, 215 W Janss Road, Thousand Oaks, CA USA

**Keywords:** Tricuspid Valve Infective Endocarditis, ESBL *E.coli*, AV block, Epicardial pacing

## Abstract

**Background:**

Infective endocarditis (IE) caused by non-HACEK gram-negative organisms accounts for fewer than 2% of cases, and endocarditis due to extended-spectrum β-lactamase (ESBL)–producing Escherichia coli is rare. Right-sided involvement, particularly isolated tricuspid valve infective endocarditis (TVIE), and associated atrioventricular (AV) conduction abnormalities are rarely reported, creating diagnostic and management challenges, especially when pacing is required.

**Case presentation:**

A 75-year-old Caucasian man with pre-existing right bundle branch block presented with syncope one week after hospitalization for ESBL E. coli bacteremia treated with intravenous ertapenem. Electrocardiography now revealed progressive AV conduction delay with intermittent high-degree AV block. Transthoracic echocardiography was nondiagnostic, while transesophageal echocardiography identified a tricuspid valve vegetation without abscess despite antimicrobial therapy. Progressive high-grade AV block developed despite appropriate antimicrobial therapy, necessitated urgent epicardial pacing due to contraindication of transvenous systems in active infection. He completed a six-week course of ertapenem with subsequent resolution of the tricuspid valve vegetation on follow-up imaging. After infection clearance, a transvenous dual-chamber pacemaker was successfully implanted.

**Conclusions:**

This case highlights an uncommon presentation of ESBL-producing E. coli TVIE complicated by advanced AV block. It underscores the importance of early transesophageal imaging in suspected right-sided endocarditis, awareness of conduction disturbances as a marker of disease severity, and the role of epicardial pacing as a safe bridging strategy when permanent transvenous systems are contraindicated during active infection.

**Graphical Abstract:**

**Figure:**
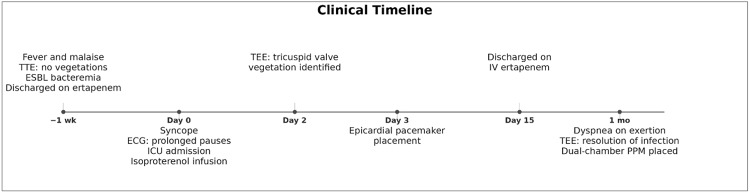

**Supplementary Information:**

The online version contains supplementary material available at 10.1186/s13019-026-04460-8.

## Background

Infective endocarditis (IE) is most commonly caused by gram-positive organisms, particularly *Staphylococcus aureus* and viridans group streptococci, while gram-negative bacteria remain an uncommon etiology. In a large prospective multinational cohort, non-HACEK gram-negative bacilli accounted for only 1.8% of all definite IE cases [1]. Among these pathogens, endocarditis caused by extended-spectrum β-lactamase (ESBL)-producing organisms has been described only sporadically in the literature [2–4]. Consequently, the clinical characteristics and optimal management of ESBL-producing *E. coli* endocarditis remain incompletely defined, creating diagnostic uncertainty and challenging management decisions, particularly when atypical manifestations such as conduction system abnormalities are present.

ESBL-producing Escherichia coli has emerged as a significant cause of bloodstream infection, particularly among older adults with healthcare exposure [5]; however, its ability to cause native valve infective endocarditis remains poorly understood [2]. The development of infective endocarditis generally requires endothelial injury, followed by bacterial adhesion, colonization, and vegetation formation. Unlike traditional endocarditis pathogens such as Staphylococcus aureus, Escherichia coli possesses relatively limited intrinsic capacity for valvular adherence, which may partially explain its infrequent involvement in native valve endocarditis [2]. Nevertheless, certain extraintestinal pathogenic E. coli (ExPEC) strains possess virulence factors, including adhesins, biofilm-forming capabilities, and immune-evasion mechanisms, which may facilitate endovascular infection in susceptible hosts [6]. 

Reported cases of ESBL-associated infective endocarditis most commonly involve left-sided valves, particularly the mitral valve, and are frequently complicated by embolic events or require surgical intervention [2, 7]. Isolated right-sided involvement is encountered less frequently and is typically associated with predisposing factors such as intravenous drug use, intracardiac devices, or central venous catheters [8]. Right-sided infective endocarditis accounts for only 5–10% of all cases of infective endocarditis, and the lower incidence of isolated right-sided disease has been attributed in part to differences in right-sided hemodynamics and endothelial stress compared with left-sided cardiac structures [8]. In addition, perivalvular extension of infection and abscess formation, which are well-recognized complications of left-sided infective endocarditis, are infrequently reported in isolated tricuspid valve infective endocarditis [8]. Consequently, isolated tricuspid valve infective endocarditis in patients without traditional risk factors remains an unusual clinical presentation, making the present case notable both for its valve location and associated conduction abnormality.

## Case presentation

A 75-year-old Caucasian male with pre-existing right bundle branch block and one year history of Whipple procedure with biliary stent placement presented four days after discharge from a recent hospitalization for sepsis due to extended-spectrum β-lactamase (ESBL) *Escherichia coli* bacteremia, treated with intravenous ertapenem. He reported dizziness following a syncopal episode that occurred while sitting in the passenger seat of his car. The loss of consciousness lasted approximately 30 s and was preceded by visual changes and mild dizziness. The patient otherwise led an active lifestyle, regularly playing golf, and had no other acute complaints.

**Fig. 1:**
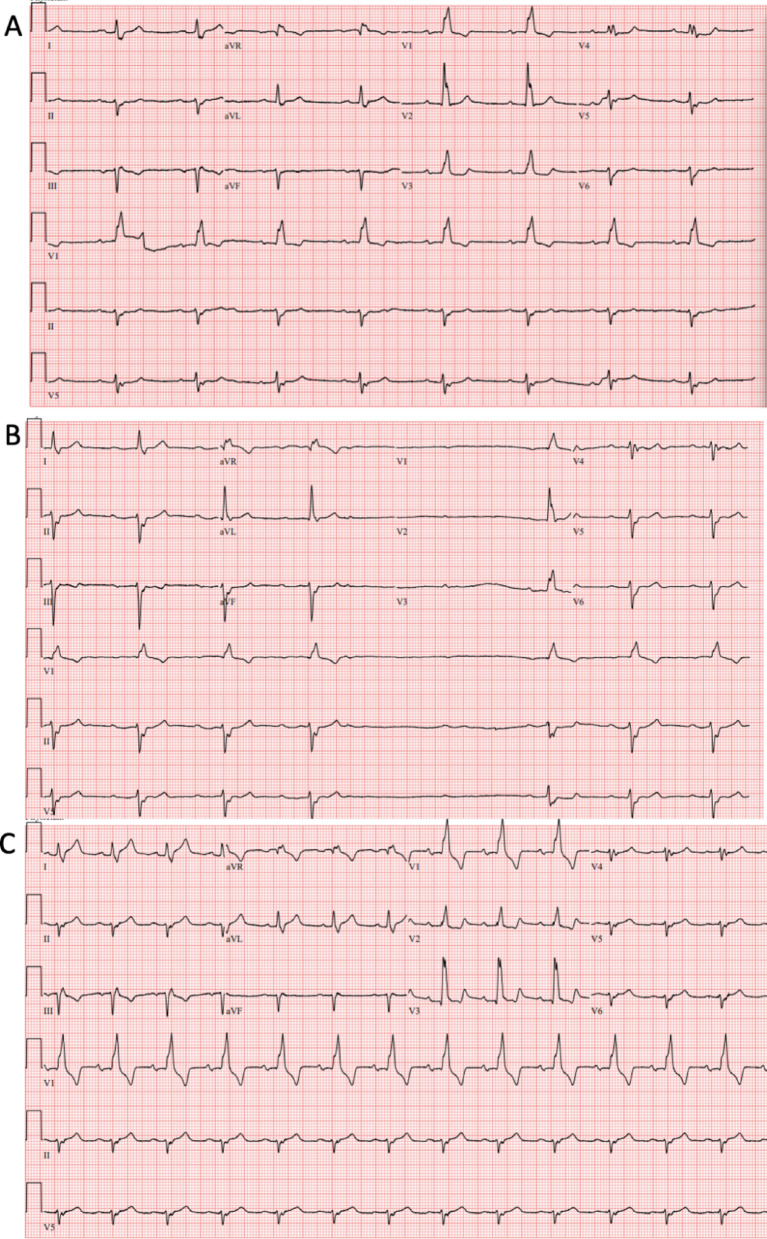
A: baseline rhythm (PR 242) B: At presentation (PR 344) C: Paced rhythm (PR 188)

On examination, he was hemodynamically stable with normal vital signs. Cardiac evaluation revealed a regular rate and rhythm, without murmurs, rubs, or gallops. Pulmonary examination was unremarkable. Electrocardiography demonstrated sinus bradycardia with first-degree atrioventricular (AV) block and intermittent pauses, left axis deviation, and pre-existing right bundle branch block **(**Fig. 1**).** Telemetry monitoring revealed intermittent high-degree AV block.

**Fig. 2:**
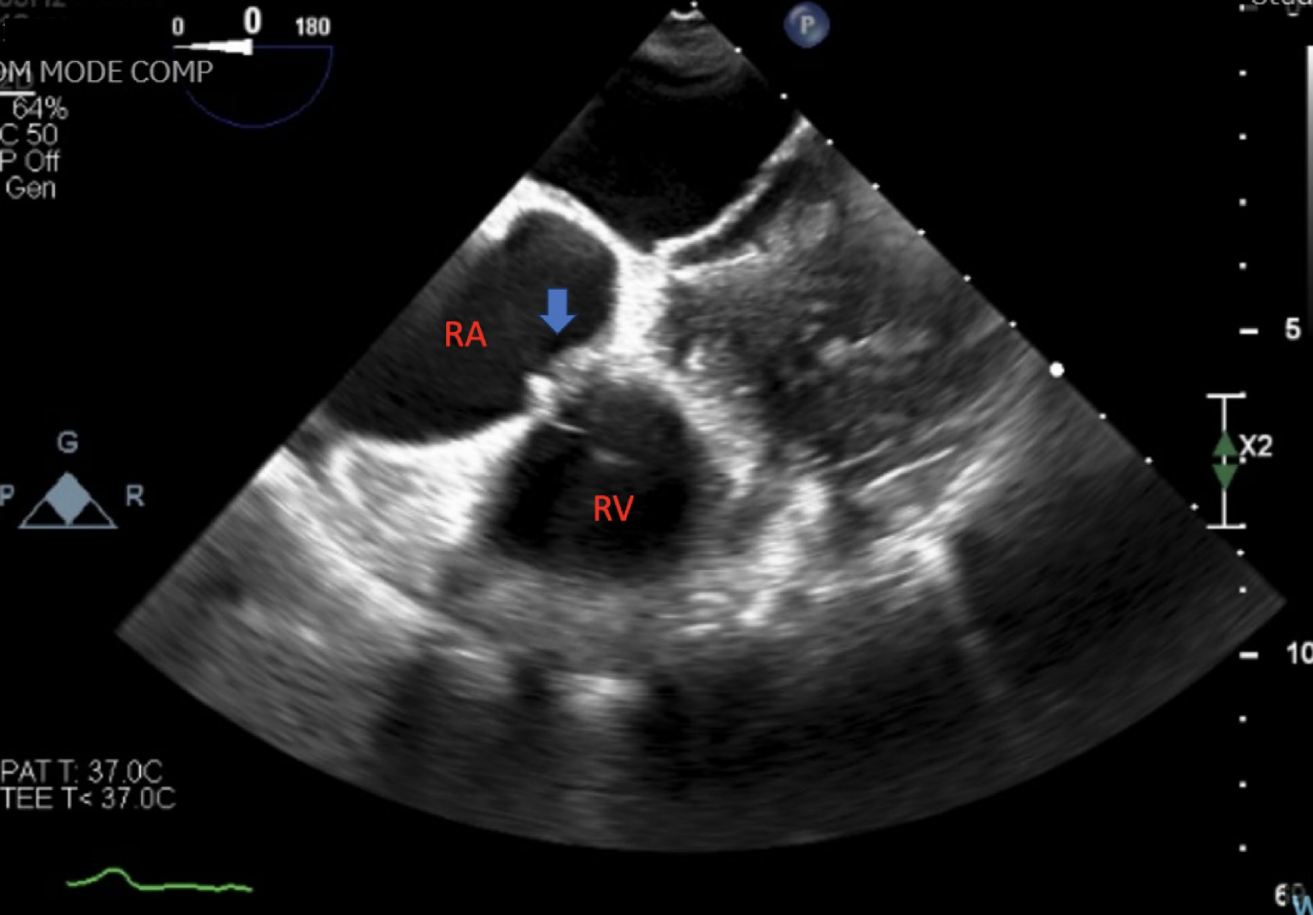
Non-standard TEE view demonstrating vegetation on tricuspid valve (blue arrow)

Laboratory evaluation revealed normal white blood cell count and hemoglobin. Central metabolic panel showed mild hyperkalemia (potassium of 5.3) and mildly elevated hepatic enzymes (Aspartate Aminotransferase 40, Alkaline Phosphatase 130) with preserved renal function. Blood cultures at this presentation were negative, however were positive for ESBL *E. coli* in admission one week prior. Transthoracic echocardiogram (TTE) demonstrated normal left ventricular function without valvular abnormalities; however, the tricuspid valve was poorly visualized. Subsequent transesophageal echocardiogram (TEE) revealed a mobile mass sitting on tricuspid valve, close to septum, about 1 cm long with flagellating distal end on atrial side of the valve with no evidence of abscess formation. Mild tricuspid regurgitation was also noted **(**Fig. 2**).**

Differential diagnosis for his syncope included cardiac causes (progressive AV block), dehydration, neurological etiologies, and medication-related adverse effects (ertapenem). Contributing factors for AV block included age-related degenerative disease, coronary ischemia, and metabolic derangements such as hyperkalemia or hypothyroidism. Investigations from his prior admission revealed ESBL bacteremia in both aerobic and anaerobic cultures, with sensitivity to meropenem and piperacillin-tazobactam. As part of the evaluation, a transthoracic echocardiogram was performed and demonstrated no evidence of valvular vegetations or infective endocarditis. Infectious disease consultation at that time failed to identify a definitive source; urinalysis was normal. Bacteremia was attributed to mild cholangitis with polymicrobial bloodstream infection. Of note at the time, vitals notable for elevated temperate of 101.5 Fahrenheit and mild jaundice. Labs notable for elevated direct bilirubin at 3.2 mg/dL, aspartate amiontransferase 76 U/L, alanine amiontransferase 88 U/L, alkaline phosphatase 153 U/L.

**Fig. 3:**
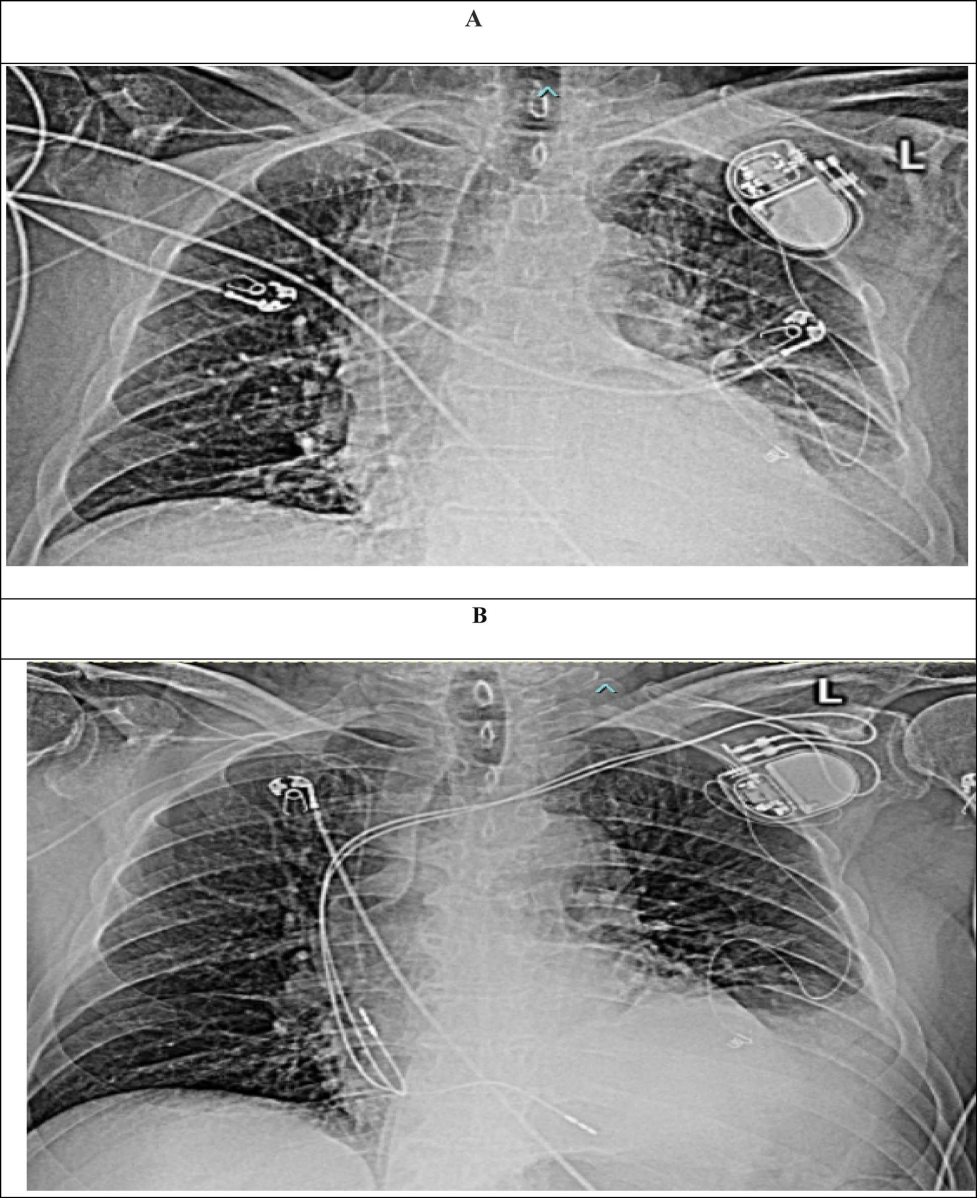
**A**: Chest radiograph demonstrating a temporary epicardial ventricular pacing lead with generator positioned in the left subpectoral region. **B**: Chest radiograph demonstrating endocardial atrial and ventricular leads in appropriate position functioning as a dual-chamber pacemaker

Due to the persistence of his symptoms in this current admission, he was admitted to the cardiac care unit and started on an isoproterenol infusion. Given the contraindication of transvenous right ventricular pacemaker placement in active infective endocarditis, cardiothoracic surgery performed urgent placement of a temporary epicardial ventricular lead via mini-thoracotomy **(**Fig. 3a**).** The lead was tunneled to a left subpectoral pocket containing a dual-chamber pacemaker, with the atrial lead intentionally capped. The patient experienced improved hemodynamic and mental status post-procedure, with only mild surgical-site pain.

The post-operative course was complicated by left pleural effusion and atelectasis, which was resolved with supportive management and continuation of intravenous antibiotics. The patient completed a six-week course of ertapenem and was discharged with close outpatient follow-up. One month later, repeat TEE demonstrated complete resolution of the tricuspid valve vegetation. At that time, new endocardial atrial and ventricular leads were placed, converting the system to a fully functional dual-chamber pacemaker **(**Fig. 3b**).**

## Discussion

Though the pathogenesis of ESBL-producing *Escherichia coli* endocarditis is not well understood, recent studies have aimed to characterize virulence factors within phylogenetic group B2. This group has a strong association with extraintestinal pathogenic *E. coli* (ExPEC), which are known to cause severe invasive infections [9]. The most notorious member of this group, *E. coli* clone ST131, has been identified as an emerging global threat due to its extensive repertoire of virulence and antimicrobial resistance genes [10]. Capsule formation, adhesion factors, and biofilm formation and maturation facilitate immune evasion and may create favorable conditions for the development of infective endocarditis [11]. Plasmid-associated acquired resistance genes (ARGs), such as CTX-M, as seen in our case, further limit effective antimicrobial options and may contribute to bacterial persistence. The combined presence of virulence factors and antimicrobial resistance genes presents significant challenges in clinical practice, often necessitating the use of more potent antibiotics. In this patient, the virulence and resistance profile of the organism likely contributed to persistent infection and may explain why epicardial pacing was required rather than antibiotics alone.

To our knowledge, only one other case — reported by Fordyce et al. — has described tricuspid valve endocarditis due to extended-spectrum β‑lactamase-producing *Escherichia coli* complicated by complete heart block [12]. While the Fordyce case and several other reports of TVIE complicated by atrioventricular conduction delay suggest resolution of block with antibiotics, conduction abnormalities in right-sided endocarditis remain exceedingly rare. In our patient, despite one week of appropriate antimicrobial therapy, there was progressive AV conduction delay with PR interval prolongation and intermittent pauses, including episodes of third-degree heart block. Because of his pre-existing conduction disease and symptomatic progression to high-grade atrioventricular block despite ongoing antimicrobial therapy, the decision was made to proceed with temporary epicardial pacing. This approach is consistent with evidence that baseline conduction delays such as first-degree AV block in infective endocarditis are associated with progression to high-degree AV block, reflecting more severe or invasive disease and justifying early pacing intervention [13]. 

In patients with infective endocarditis (TVIE) complicated by new or worsening atrioventricular (AV) block, surgical intervention is indicated especially if the infection is complicated by valve dysfunction, including a resistant organism, persistent infection and relapsing prosthetic valve endocarditis [14]. In aortic or mitral valve infective endocarditis, new or worsening atrioventricular conduction abnormalities often raise concern for perivalvular extension of infection and may prompt consideration of early surgical intervention [15]. Although the atrioventricular node lies within Koch’s triangle and is anatomically adjacent to the septal leaflet and annulus of the tricuspid valve, conduction disturbances remain an uncommon manifestation of isolated tricuspid valve infective endocarditis. [16, 17] This may reflect the lower frequency of perivalvular extension and abscess formation in right-sided disease compared with aortic valve endocarditis. In the absence of abscess formation, conduction abnormalities in tricuspid valve infective endocarditis may instead result from local inflammation, edema, or transient involvement of adjacent conduction tissue.

Case reports support that antibiotic therapy alone can lead to resolution of AV conduction disturbances in TVIE and allows surgical intervention to be deferred. For example, Martínez‑Urueña et al. described a patient with tricuspid valve vegetations and transient trifascicular block who improved with antibiotic therapy and temporary pacing, without surgical intervention [16]. Similarly, Singh and Kalathiya reported reversible complete heart block in tricuspid valve endocarditis managed successfully with antibiotics, highlighting that conduction abnormalities may resolve once inflammation and bacteremia are controlled [17]. In our reported case, the decision was made to proceed with epicardial pacing rather than continue antibiotic therapy as the patient was severely symptomatic from the arrhythmia and had baseline AV block. An alternative strategy would have been semipermanent externalized right ventricular pacing using an active-fixation transvenous lead connected to an external pulse generator. This approach has been reported as a safe and effective option for patients requiring prolonged temporary pacing and offers advantages including improved lead stability and patient mobility compared with conventional temporary pacing systems [18]. However, in the present case, the vegetation involved the tricuspid valve near the septal leaflet, and placement of a transvenous RV lead would have required traversing the infected valve. Following multidisciplinary discussion, temporary epicardial pacing was therefore selected to avoid introducing additional transvenous hardware across the site of active infection until completion of antimicrobial therapy and documented resolution of the vegetation. Of note, Leadless pacing systems such as the Micra transcatheter pacemaker may represent an alternative strategy in patients requiring pacing in the setting of infection because they eliminate the need for a subcutaneous pocket and transvenous leads and have demonstrated low rates of device-related infection. However, evidence supporting their use in patients with active native tricuspid valve infective endocarditis is limited.

While this case illustrates a rare clinical presentation of ESBL-producing E. coli endocarditis with conduction abnormalities, several limitations should be considered. Blood cultures were negative in this presentation; however, the patient had received daily intravenous ertapenem therapy for ESBL-producing E. coli bacteremia one week prior to admission, which may have contributed to culture negativity. A previous report described a similar scenario in which blood cultures were negative, but tissue cultures from a tricuspid valve replacement revealed ESBL-producing *Escherichia coli* as the causative pathogen [7]. Another limitation in our case is the patient’s pre-existing atrioventricular (AV) block. Although age-related degenerative conduction disease could have contributed to worsening AV conduction, it is notable that the patient developed third-degree AV block and progressive PR prolongation coinciding with the endocarditis. While we cannot definitively prove causation, the temporal relationship between the infection and conduction abnormalities supports the likelihood that the endocarditis contributed to the conduction disturbance, ultimately prompting the placement of an epicardial pacemaker.

## Conclusion

This case highlights the importance of maintaining a high index of suspicion for infective endocarditis in patients with persistent or recent gram-negative bacteremia who develop new or worsening conduction abnormalities, even in the absence of traditional risk factors for right-sided disease. Early transesophageal echocardiography was essential for establishing the diagnosis when transthoracic imaging was nondiagnostic and directly influenced management. Furthermore, this case demonstrates that symptomatic high-grade atrioventricular block occurring in the setting of active tricuspid valve infective endocarditis may require individualized pacing strategies when transvenous systems are undesirable. Recognition of these uncommon presentations may facilitate earlier diagnosis, multidisciplinary intervention, and improved patient outcomes.

## Supplementary Information

Supplementary Material 1.

## Data Availability

No datasets were generated or analysed during the current study.

## Abbreviations

IE: Infective Endocarditis
TVE: Tricuspid Valve Infective Endocarditis
ESBL: Extended-Spectrum β-Lactamase
*E. coli*: Escherichia coli
AV: Atrioventricular
TTE: Transthoracic Echocardiography
TEE: Transesophageal Echocardiography
RBBB: Right Bundle Branch Block
ExPEC: Extraintestinal Pathogenic* Escherichia coli*
ST131: Sequence Type 131
ARGs: Antimicrobial Resistance Genes
CTX-M: Cefotaximase-Munich (β-lactamase enzyme family)
